# Large Language Model-Based Interactive Code Generation for Developing a 3D Eye Movement Schematic

**DOI:** 10.7759/cureus.107791

**Published:** 2026-04-27

**Authors:** Ichiro Hamasaki, Keiko Kunimi, Kiyo Shibata, Goseki Toshiaki

**Affiliations:** 1 Ophthalmology, Lino Eye Clinic, Okayama, JPN; 2 Ophthalmology, Kanagawa Dental University Yokohama Clinic, Kanagawa, JPN; 3 Ophthalmology, International University of Health and Welfare, Atami Hospital, Shizuoka, JPN

**Keywords:** artificial intelligence, computer simulation, eye movements, large language models, medical education, strabismus, three-dimensional imaging

## Abstract

Introduction

Understanding the three-dimensional (3D) geometric relationship between extraocular muscles and the globe is essential for strabismus management. Conventional educational tools are static, and existing 3D biomechanical software requires highly specialized skills, making routine clinical use difficult. Furthermore, image-generating artificial intelligence (AI) frequently produces anatomically incorrect outputs (hallucinations). This study aimed to develop a structurally coherent, interactive 3D eye movement schematic as a proof-of-concept, using the coding capabilities of a large language model (LLM).

Methods

We used an LLM to generate web-based 3D schematic code (HTML and JavaScript/Three.js) exclusively through natural language dialogue. To prevent anatomical errors, we explicitly defined anatomical parameters based on standard literature (e.g., 12-mm scleral radius) and employed mathematical constraints, including quaternions for rotation and spherical linear interpolation for muscle paths, within the prompts. The generated code was rendered in a web browser, and an iterative process of prompt refinement and debugging was conducted until two board-certified ophthalmologists confirmed the schematic's structural validity.

Results

A functional, interactive 3D eye movement schematic was successfully developed. In our 10-trial evaluation, generating an acceptable schematic required an average of 7.4 prompt inputs per session. While complex spatial instructions had a lower success rate (40%) due to AI hallucinations, iterative prompt repetition and specific local debugging instructions resolved these issues. The final schematic provided a structurally coherent representation of the globe, the four rectus muscles, and the annulus of Zinn. It featured a slider interface enabling real-time, kinematic visualizations of eye rotations, muscle deformations, and optic nerve bending without structural failure.

Conclusions

Translating anatomical descriptions into mathematical spatial logic via LLMs enables the creation of structurally sound 3D medical schematics. This logical spatial construction approach democratizes the development of interactive educational tools. It allows healthcare providers without programming expertise to intuitively generate customizable 3D educational materials for patient consultations and foundational medical education through natural language dialogue.

## Introduction

Understanding the three-dimensional (3D) geometric relationship between extraocular muscles and the globe is essential for strabismus management. However, conventional 2D illustrations and physical models inadequately convey dynamic eye movements and the spatial alterations resulting from surgery. While existing biomechanical models [1-3] and 3D computer graphic (CG) educational tools [4,5] offer high accuracy, their operation requires specialized knowledge or steep learning curves, making routine clinical customization impractical.

Concurrently, although image-generating artificial intelligence (AI) is increasingly utilized, it frequently produces anatomically incorrect extraocular structures - a phenomenon known as AI "hallucination" - rendering it unsuitable for precise medical education [6-8]. Currently, a practical tool that allows healthcare providers without CG skills to intuitively create structurally coherent, dynamic 3D schematics is lacking.

To address this gap, we focused on the coding capabilities of large language models (LLMs). We devised a method to generate web-based 3D eye movement simulation code (JavaScript/Three.js) using solely natural language instructions. While not replicating complex nonlinear mechanics like the active pulley system, this model dynamically represents fundamental anatomical structures - from the annulus of Zinn to the Spiral of Tillaux - and the associated globe rotations. As a proof-of-concept, this paper demonstrates that healthcare providers without programming knowledge can easily develop customizable 3D models for patient and student education, highlighting a practical application of AI in medical communication.

## Materials and methods

To verify the capability of a large language model (LLM) to generate structurally coherent 3D schematics, we utilized Gemini 3.1 Pro (Google LLC, Mountain View, CA) to develop an interactive eye movement schematic as a proof-of-concept. To evaluate its feasibility in a standard clinical setting, the LLM was accessed exclusively through its default consumer web interface without any application programming interface (API) parameter adjustments. Through natural language dialogue, the LLM was tasked with generating HTML and JavaScript code using the Three.js (WebGL) library. The generated code was rendered in an online editor (CodePen, CodePen, Inc., Bend, OR) for immediate visual feedback. Since the output consists of standard HTML and WebGL-based JavaScript, the schematic runs directly on modern web browsers without specific constraints and does not require high-end graphics hardware; all development and rendering in this study were smoothly conducted on a standard personal computer (11th-generation Intel Core i5).

To mitigate AI hallucinations while acknowledging the probabilistic nature of LLMs, we specified precise shapes and explicitly defined anatomical and mathematical rules. Based on standard literature [9,10], ocular components such as the sclera (12 mm radius) and cornea (7.7 mm radius) were strictly parameterized, alongside mathematical constraints like spherical linear interpolation for muscle paths. Since the success of 3D construction requires visual confirmation, two board-certified ophthalmologists (IH and KK) conducted an iterative prompt-evaluate-refine cycle using separate personal computers to cross-verify the results during development. They evaluated the rendered schematics based on geometric proportions, muscle paths, and dynamic structural integrity. When the LLM failed to produce the intended structures, we debugged it using natural language by providing corrective prompts based on observed visual errors. We continued this trial-and-error dialogue until the experts reached a consensus that the generated schematic visually aligned with clinical educational standards. Furthermore, to quantify the required iterations and success rates, we conducted 10 independent evaluation trials of the entire workflow, recording the number of initial and corrective prompts needed to achieve an acceptable geometry. To enable reliable reproduction by other users, the exact texts of the core prompts and the specific debugging steps are fully documented in the Results section.

## Results

In the initial phase, we input Prompt 1 into the AI tool to construct a base 3D model.

Prompt 1: "You are a researcher who is an expert in the anatomy of the eye. Create a 3D model of the 'eyeball' and '4 rectus muscles' to explain to beginners, using HTML (including JavaScript) that runs in a browser. Define and strictly enforce the Y-axis as the visual axis, with +Y as anterior (corneal side) and -Y as posterior (optic nerve side). Do not display the XYZ axes. Set position the 3D model so the cornea is facing the front. Allow the user to freely rotate, zoom, and pan using the mouse."

**Figure 1:**
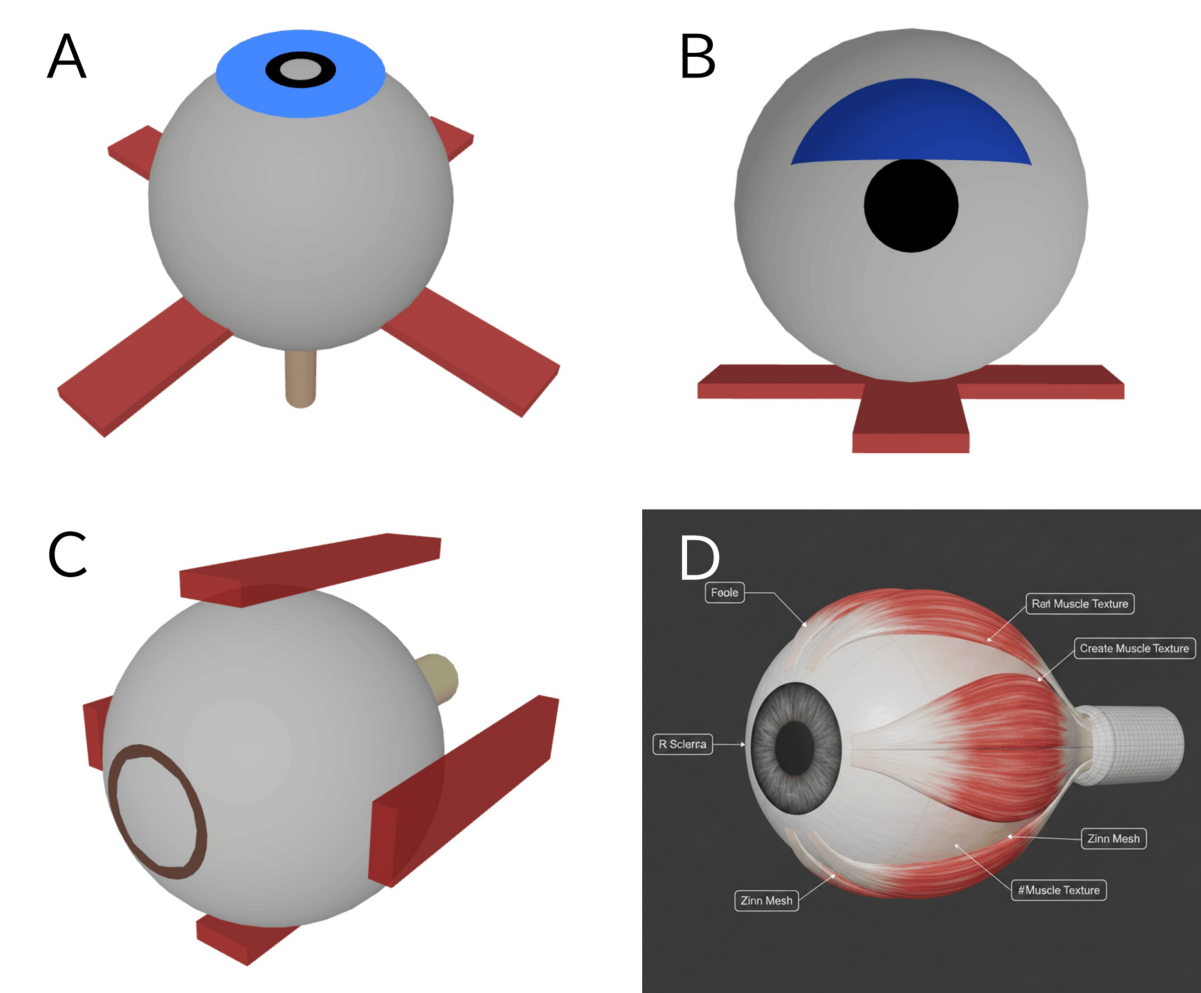
Limitations of generative AI in eye modeling. (A) Simple shapes resulting from the initial prompt. (B, C) Similar abstracted shapes produced after repeated attempts. (D) An anatomically incorrect 2D illustration demonstrating AI hallucination. Panels (A)-(C) display 3D models rendered from HTML/JavaScript code generated by Gemini 3.1 Pro (Google LLC, Mountain View, CA). Panel (D) was generated using the image generation tool, Nano Banana Pro (Google LLC).

The code generated from Prompt 1 produced a simple combination of basic shapes, such as spheres, cylinders, and cuboids, lacking practical utility (Figure 1A). Repeated attempts yielded similar geometric shapes (Figure 1B, C). As a control, we instructed an image-generating AI (Nano Banana Pro, Google LLC, Mountain View, CA) to create an illustration of the eye and four rectus muscles. This resulted in anatomically incorrect images (Figure 1D). Despite repeated requests for correction, we confirmed that creating and modifying precise anatomical structures using the image-generation approach is impractical.

Next, we input Prompt 2 to specify the dimensions and spatial arrangement of the ocular components.

Prompt 2: "Sclera: A sphere with a 12 mm radius. An opening with a 6 mm radius in the +Y direction (anterior). Cornea: A spherical cap with a 7.7 mm radius. White color with 10% opacity. Place it tightly against the opening. Iris/Pupil: A flat brown iris at the sclera opening, with a black pupil of 1.5 mm radius in the center. Optic nerve: A cylinder with a 2 mm radius and 30 mm length, light gray. Extending in the -Y direction (posterior). Annulus of Zinn (Common tendinous ring): Placed around the optic nerve at the furthest position from the eye (around Y=-30 mm). Shape: A 'white, single polygon sheet (DoubleSide rendering) tube (cylinder)' with a thickness 1.2 times the diameter of the optic nerve. It should be a tube with depth, not a flat ring."

**Figure 2:**
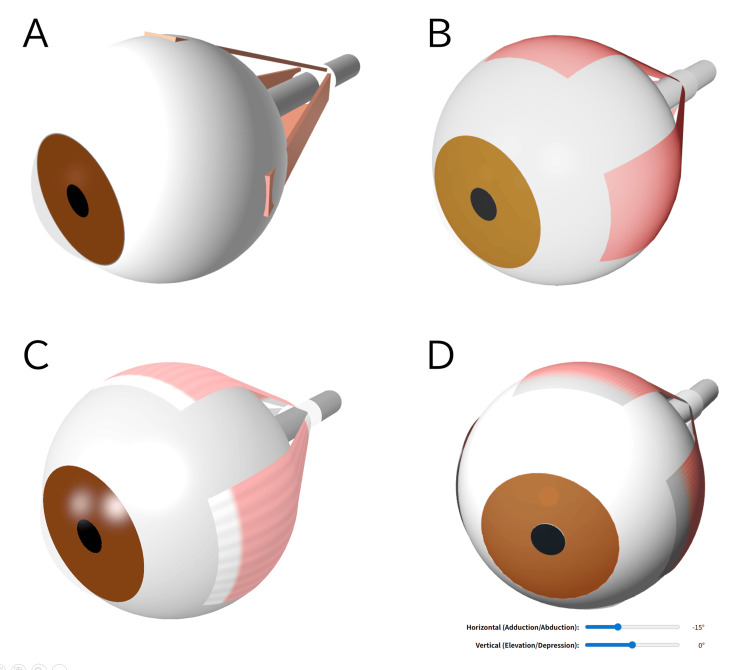
Iterative development process of the 3D eye schematic. (A) Basic ocular components generated from anatomical parameters. (B) Integration of the anatomical paths of the four rectus muscles. (C) Visual improvements and structural refinements. (D) Implementation of an interactive slider interface for dynamic eye movement simulation. All panels display 3D models rendered from HTML/JavaScript code generated by Gemini 3.1 Pro (Google LLC).

Inputting these anatomical values [9,10] structured the ocular components, bringing the model closer to the target 3D schematic (Figure 2A). However, because the four rectus muscles were insufficiently defined at this stage, their shape and path remained unnatural.

To reproduce the anatomical path of the extraocular muscles in 3D space, we input Prompt 3.

Prompt 3: "4 rectus muscles (Superior, Inferior, Medial, Lateral Rectus) - Shape: A 'single polygon sheet (DoubleSide rendering)' with no thickness. Width: 10 mm at the insertion (sclera side). Converging in a fan shape towards the annulus of Zinn. Course: (1) Connect the origin (annulus of Zinn) to the 'point of tangency' where it touches the eye surface with a straight line (plane) representing the shortest distance in the air. (2) From the tangency point to the insertion, the muscle must stick tightly along the curve of the eye (using Spherical Linear Interpolation / Slerp). (3) Place the muscle slightly away from the sclera so it does not bury/clip into it. (4) Calculate mathematically so the muscles do not twist or fold. The 4 rectus muscles attach in an arc along the curved surface of the sclera at the position of the Spiral of Tillaux. They also attach in an arc to the 4 divided quadrants of the anterior circular edge of the annulus of Zinn."

Prompt 3 reproduced the natural anatomical course of the extraocular muscles along the spherical surface of the globe, advancing the schematic's structure (Figure 2B).

Finally, to improve the visual quality for educational purposes and implement dynamic simulation features, we sequentially input Prompts 4 and 5.

Prompt 4: "Color gradient: Make 10% of both ends of the muscle (attachments at the sclera and annulus of Zinn) 'white (tendon)' and the central part 'light red (muscle belly)', and smoothly interpolate between them. Striation pattern: Apply a schematic striped pattern across the entire muscle to show muscle fibers along the direction of the muscle course, using a schematic rather than realistic color scheme. There are no muscle fibers in the tendon parts. To avoid confusion if the sclera and tendons are the same color, make the tendons a slightly light gray. Fix the light source at the top left from the camera's perspective and create a reflection image (highlight) on the cornea."

Prompt 4 improved the visibility of the globe and rectus muscles, completing the intended 3D schematic (Figure 2C). Further visual adjustments were easily achieved using individual descriptive prompts.

Prompt 5: "Add slide bars to control the adduction/abduction and elevation/depression movements of the eye, and replicate these movements with the eye and the 4 rectus muscles. The annulus of Zinn must remain stationary during eye movement, and the optic nerve passes through it. Express the optic nerve bending softly at the base of the eye, and ensure the optic nerve does not protrude outside the muscle cone."

Prompt 5 enabled a dynamic simulation. Globe rotation, deformation of the four rectus muscles, and bending of the optic nerve synchronized continuously with the slider user interface (UI) operations without any structural errors (Figure 2D).

**Figure 3:**
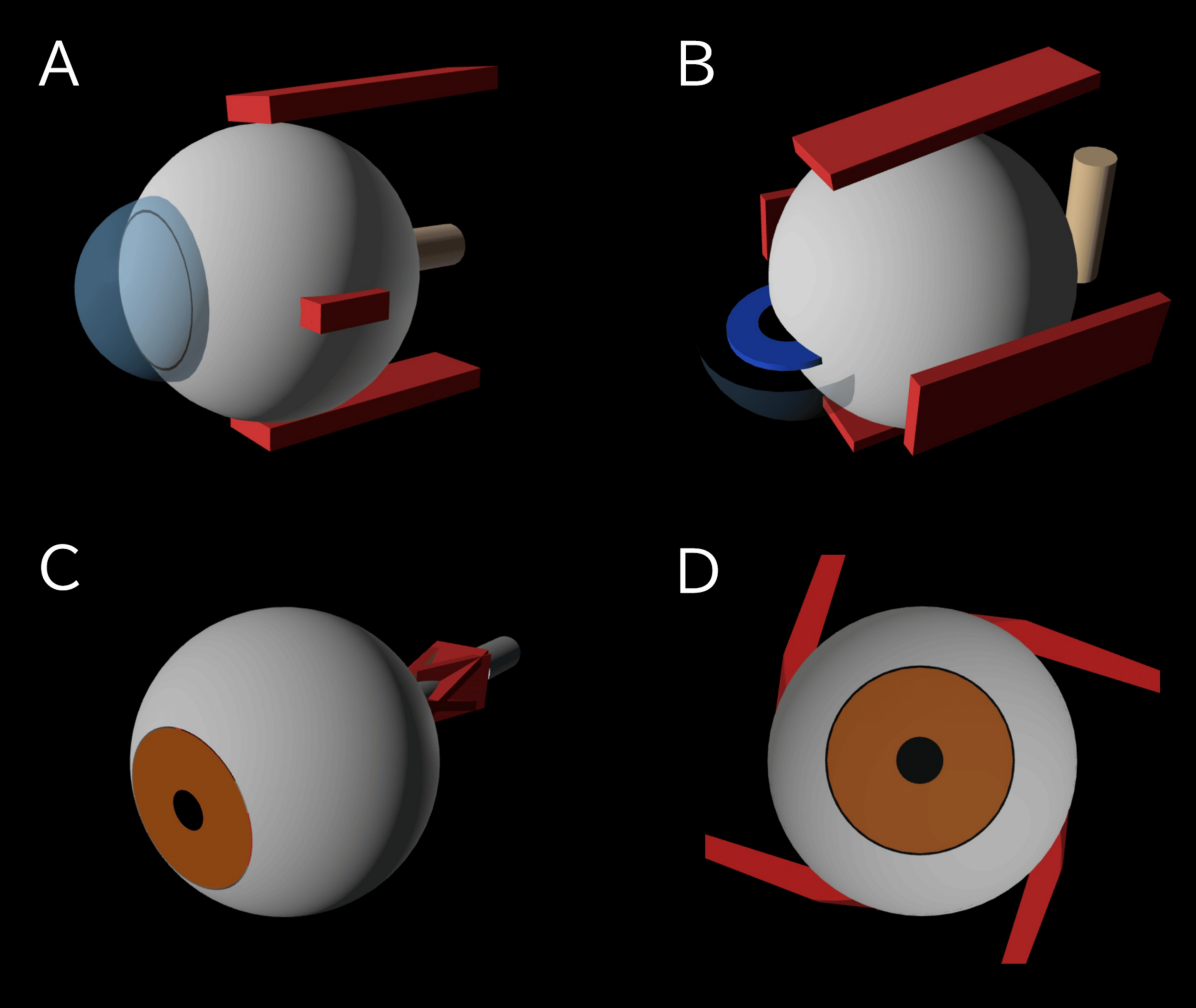
Structural failures in the 3D eye schematic generation. (A) Cuboid muscles. (B) Misaligned cornea and optic nerve. (C) Muscles excessively buried in the sclera. (D) Deviated muscle paths. All panels display initial geometric errors rendered from HTML/JavaScript code generated by Gemini 3.1 Pro (Google LLC).

Throughout the generation process, initial prompts did not consistently yield structurally coherent representations on the first attempt (Figure 3). Initial outputs often lacked biomechanical constraints, resulting in unintended geometric errors. Observed anomalies included cuboid muscles (Figure 3A), axial misalignment of the cornea and optic nerve (Figure 3B), muscles excessively buried in the sclera (Figure 3C), deviated muscle paths (Figure 3D), and the optic nerve detaching from the orbital apex during rotation.

To resolve these anomalies, we refined the generated schematic using two main approaches. First, we repeatedly submitted the same prompts [11,12]. This strategy leveraged the probabilistic nature of the LLM's output to identify a baseline schematic with fewer spatial errors. Second, we provided targeted corrective prompts to address persistent structural errors. For example, we added explicit natural language instructions such as, "Fix the 4 rectus muscles burying into the sclera," or "Prevent the optic nerve from protruding from the annulus of Zinn during eye movement." The LLM successfully translated these natural language corrections into updated mathematical constraints within the generated code, progressively enhancing the structural coherence of the schematic.

To quantify the required iterations and the frequency of structural failures, we conducted 10 independent trials of the entire generation process, sequentially executing Prompts 1 through 5. In each trial, if the output was structurally acceptable, we proceeded to the next prompt. If AI hallucinations or unexpected structures appeared, we applied corrective prompts iteratively until an acceptable geometry was achieved. To evaluate the efficiency of each step, the success rate was calculated by dividing the number of successful completions (n=10) by the total number of attempts, which included the initial command and all corrective iterations.

The observed success rates were 77% for Prompt 1 and 100% for Prompt 2, with the latter requiring no corrections. Prompt 3, which involved complex mathematical constraints for 3D muscle paths, exhibited a lower success rate of 40%. Prompts 4 and 5 achieved 83% and 71%, respectively. The lower success rate for Prompt 3 was primarily due to a higher frequency of AI hallucinations producing unexpected spatial configurations, necessitating multiple corrective iterations - although some trials succeeded on the first attempt. Overall, completing the five-step schematic generation required an average of 7.4 prompt inputs per session. This demonstrates that typically only two to three corrective instructions were needed alongside the five base prompts to finalize the model.

## Discussion

The results demonstrate that healthcare professionals without programming knowledge can build structurally coherent, interactive 3D eye movement schematics through natural language dialogue with an LLM. In our control test, image-generating AI produced anatomically incorrect images. This supports the concerns raised by Sallam [13] regarding generative AI in medical education, highlighting the topological limitations and hallucination risks of pixel-based generative models. Furthermore, as discussed by Thirunavukarasu et al. [14], controlling plausible but incorrect outputs remains a primary challenge in applying generative AI to medicine. While image-generating AI probabilistically creates eye-like textures, our approach of having an LLM write HTML and JavaScript (Three.js) enforces strict mathematical constraints, such as vertex coordinates in 3D space. This methodology ensures the structural consistency and reproducibility required for medical education tools.

A notable aspect of the schematic construction process was the necessity of prompt engineering. Anatomical terminology required translation into geometric and mathematical definitions, as initial abstract instructions failed to produce practical schematics. However, by strictly defining scleral and corneal dimensions based on standard anatomical and optical values [9,10], and mathematically describing muscle wrapping as spherical linear interpolation, we successfully reproduced natural muscle paths. This underscores that LLMs lack inherent visual or spatial comprehension of anatomical structures; instead, they translate user language into mathematical spatial logic. Importantly, when the LLM initially failed to generate perfect code, users could correct visual errors by describing them in natural language. An iterative process of repeated dialogue consistently facilitated the convergence of the schematic to the intended geometry [11,12]. This demonstrates that intuitive debugging through dialogue with AI is highly feasible, even for healthcare providers unfamiliar with programming.

An additional finding of this study was the autonomous completion of anatomical parameters by the LLM. When specifying the extraocular muscle paths, we merely instructed an arc-like attachment at the Spiral of Tillaux using natural language, without inputting specific distances from the limbus (e.g., 5.5 mm for the medial rectus) as described by Apt [15]. Nevertheless, the LLM automatically implemented asymmetrical insertion coordinates. As Thirunavukarasu et al. [14] noted, medical knowledge is encoded within LLMs through pre-training. This autonomous completion of numerical values demonstrates that the LLM extracted its latent medical knowledge as mathematical parameters to construct a 3D space, rather than merely generating text. While this feature significantly reduces the user's input burden, the outputs are not always flawless. Therefore, a "human-in-the-loop" process - reviewing and correcting the generated values by human experts [14] - remains essential.

Regarding the development environment, this methodology is highly suitable for routine clinical implementation, as it requires no prior programming expertise. Because the LLM generates and debugs code through natural language dialogue, clinicians can easily adopt this approach. While we utilized external web editors like CodePen for visual validation in this study, the recent emergence of LLMs equipped with integrated code-preview features (e.g., Claude) provides further advantages. Utilizing such platforms allows clinicians to generate and modify 3D schematics in real time in front of patients without leaving the AI chat interface, providing a seamless user experience for informed consent and clinical explanations.

A limitation of this study is that the current model is a kinematic schematic rather than a dynamic simulation, reflecting the nonlinear elasticity of orbital connective tissues. Developing a precise biomechanical model would require exact coordinate data, such as those utilized in conventional 3D software [4]. Another key limitation is the lack of quantitative validation; anatomical parameters were evaluated via subjective expert consensus rather than statistical comparison with ground truth datasets. Furthermore, the probabilistic nature of LLMs introduces variability and hallucination risks. In our 10-trial evaluation, success rates fluctuated depending on task complexity. Simple shape definitions succeeded without corrections, whereas complex spatial instructions (Prompt 3) were more prone to hallucinations, reducing the success rate to 40% due to required corrective iterations. Overall, generating an acceptable schematic required an average of 7.4 prompt inputs per session. To transition this proof-of-concept into a validated clinical tool, future studies must standardize prompting protocols, incorporate rigorous quantitative benchmarking, and conduct formal evaluations by a panel of strabismus experts to rigorously assess reproducibility, anatomical correctness, and true clinical relevance.

Nevertheless, for patient consultations and foundational medical education in daily practice, this level of modeling fidelity provides substantial value in facilitating spatial comprehension. Furthermore, this interactive 3D modeling approach using LLMs has potential applications beyond ophthalmology, serving as a versatile educational tool across various medical disciplines.

## Conclusions

In conclusion, the logical spatial construction approach using LLMs democratizes the development of 3D medical educational tools, overcoming conventional financial and technical barriers. It provides a foundation for novel medical communication tools, enabling healthcare providers without specialized programming expertise to intuitively create digital educational materials.
